# Pain during ice water test distinguishes clinical bladder hypersensitivity from overactivity disorders

**DOI:** 10.1186/1471-2490-6-31

**Published:** 2006-12-27

**Authors:** Gaurav Mukerji, Janet Waters, Iain P Chessell, Chas Bountra, Sanjiv K Agarwal, Praveen Anand

**Affiliations:** 1 Peripheral Neuropathy Unit, Hammersmith Hospital and Imperial College London, UK; 2 Department of Urology, Hammersmith Hospital and Imperial College London, UK; 3 Neurology and GI CEDD, GlaxoSmithKline Research and Development Ltd, New Frontiers Science Park (North), Harlow, Essex, CM19 5AW, UK

## Abstract

**Background:**

The Bladder cooling reflex (BCR) i.e. uninhibited detrusor contractions evoked by intravesical instillation of cold saline, is a segmental reflex believed to be triggered by menthol sensitive cold receptors in the bladder wall, with the afferent signals transmitted by C fibres. The BCR is a neonatal reflex that becomes suppressed by descending signals from higher centres at approximately the time when the child gains full voluntary control of voiding. It re-emerges in adults with neurogenic detrusor overactivity as a consequence of loss of central descending inhibition, resulting from conditions such as spinal cord injury or multiple sclerosis. We have recently shown an increase of nerve fibres expressing the cool and menthol receptor TRPM8 in both overactive (IDO) and painful bladder syndrome (PBS), but its functional significance is unknown. We have therefore studied the bladder cooling reflex and associated sensory symptoms in patients with PBS and overactivity disorders.

**Methods:**

The BCR, elicited by ice water test (IWT) was performed in patients with painful bladder syndrome (PBS, n = 17), idiopathic detrusor overactivity (IDO, n = 22), neurogenic detrusor overactivity (NDO, n = 4) and stress urinary incontinence (as controls, n = 21). The IWT was performed by intravesical instillation of cold saline (0 – 4°C). A positive IWT was defined as presence of uninhibited detrusor contraction evoked by cold saline, associated with urgency or with fluid expulsion. Patients were asked to report and rate any pain and cold sensation during the test.

**Results:**

A positive IWT was observed in IDO (6/22, 27.3%) and NDO (4/4, 100%) patients, but was negative in all control and PBS patients. Thirteen (76.5%) PBS patients reported pain during the IWT, with significantly higher pain scores during ice water instillation compared to the baseline (P = 0.0002), or equivalent amount of bladder filling (100 mls) with saline at room temperature (P = 0.015). None of the control or overactive (NDO/IDO) patients reported any pain during the IWT.

**Conclusion:**

The BCR in DO may reflect loss of central inhibition, which appears necessary to elicit this reflex; the pain elicited in PBS suggests afferent sensitisation, hence sensory symptoms are evoked but not reflex detrusor contractions. The ice water test may be a useful and simple marker for clinical trials in PBS, particularly for novel selective TRPM8 antagonists.

## Background

The bladder cooling reflex (BCR) is a segmental reflex believed to be triggered by menthol sensitive cold receptors in the bladder wall, mediated by a spinal reflex pathway [1-3]. It is thought to be a neonatal reflex that becomes suppressed by descending signals from higher centres at approximately the time when the child gains full voluntary control of voiding. Thus, it is typically absent in older children (> 4 years) and adults with stable bladders [2]. The BCR re-emerges in adults with central nervous system disorders, and was originally introduced by Bors and Blinn as a provocation test to differentiate upper from lower motor neuron lesions [3] – the presence of BCR supports the diagnosis of an upper motor neuron lesion [1-4].

The BCR evoked by cooling the bladder wall by intravesical instillation of cold saline (Ice Water test), is activated at bladder volumes or pressures below threshold for the ordinary voiding reflex, suggesting that it originates from specific cold receptors rather than mechanoreceptors [1]. This hypothesis is in accord with the identification of a cool and menthol receptor TRPM8 in the urinary bladder [5]. In a recent study, we demonstrated an increased expression of TRPM8 in suburothelial nerve fibres of patients with painful bladder syndrome (PBS) and idiopathic detrusor overactivity (IDO) [6].

Previous studies have demonstrated the BCR to be most consistently present in neurogenic detrusor overactivity (NDO) [7]. It has been proposed that as a consequence of loss of central descending inhibition in conditions such as spinal cord injury or multiple sclerosis, the BCR emerges as a segmental sacral spinal reflex mediated by C fibre afferents, which may themselves be increased by sprouting secondary to bladder hypertrophy. Fall and colleagues [4] observed that a number of patients with idiopathic bladder overactivity (IDO) and a positive ice water test (IWT) later showed overt signs of neurological diseases during long-term follow-up. Thus, a positive IWT in IDO may be an early warning sign of future neurological disease.

The urinary bladder C fibres are considered to be normally inactive, and termed "silent" C-fibres [8]. Their sensitisation, sprouting or phenotypic changes, induced by pathological conditions including inflammation and hypertrophy, may be associated with functional changes such as increased urination frequency and urgency, as well as suprapubic pain [9,10]. It has been hypothesized that chronic bladder pathological states result in recruitment of C fibres, which may contribute to painful and overactive symptoms [8].

The significance of the increase of nerve fibres expressing the cool and menthol receptor TRPM8 in both overactive bladders and PBS is uncertain, with respect to the BCR and sensory dysfunction – in the absence of a superimposed bladder infection NDO is usually a painless condition, while pain predominates in PBS. We were not aware of any previous studies of the BCR in PBS. We therefore studied the BCR in patients with overactive and painful bladder syndromes, and compared the results with controls. We also recorded the symptoms associated with intravesical ice water instillation in these patients.

## Methods

### Patient selection

The BCR, elicited by the ice water test (IWT) was performed in 64 women (age range 28–65 years). The patients had painful bladder syndrome (PBS, n = 17), who met the NIDDK research criteria for interstitial cystitis [11], or idiopathic detrusor overactivity (IDO, n = 22). The control group was selected from patients undergoing urodynamic investigation for stress urinary incontinence (n = 21). In the control group, patients with overactive symptoms and/or detrusor overactivity on urodynamics were excluded from the study. The IWT was also performed in four subjects with neurogenic detrusor overactivity (NDO, with Multiple Sclerosis (n = 3) and cerebrovascular disease (n = 1)), as positive controls. In the overactive (NDO/IDO) group, patients that showed detrusor overactivity with filling volumes less than 200 mls, or with evidence of bladder outlet obstruction on routine urodynamics, were excluded from the study.

In the PBS group, Pain score was also recorded on a Visual Analogue Scale (VAS) for worst, least and average pain during the last 24 hours before the study [12] on a scale of 0 to 10 (0 – No Pain to 10 – Intolerable pain). The Mean (range) for the worst, least and average pain scores (VAS) of our PBS patients was 4.7 (2 to 9), 0.4 (0 to 3) and 3.4 (2 to 6) respectively.

The study was approved by the local research ethical committee (Hounslow and Hillingdon LREC, Reference No. 05/Q0407/62). Informed written consent was obtained from all patients before study. Normal urinalysis was established prior to the urodynamic study. All the patients underwent routine urodynamics with intravesical instillation of normal saline at room temperature up to capacity at the rate of 50 ml/min.

### Ice Water Test

**Figure 1:**
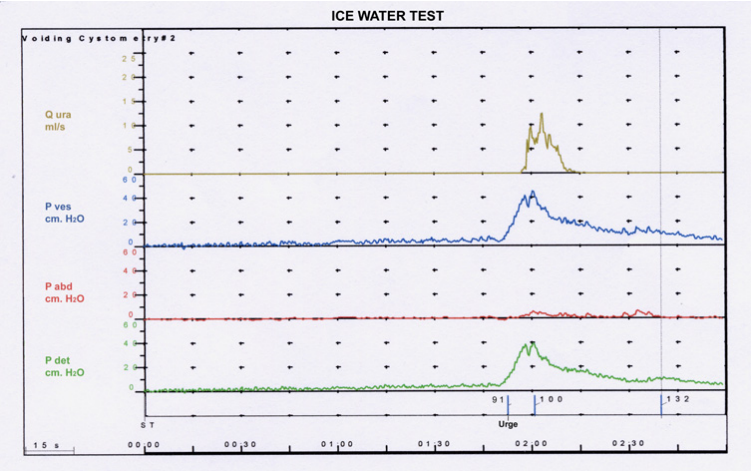
**Positive IWT in NDO patient**. Representative cystometrogram of a NDO patient showing a positive IWT associated with urgency and fluid expulsion at 88 mls filling with cold saline.

The IWT was performed at the end of routine urodynamics after emptying the bladder, by intravesical instillation of cold saline (0° to 4°C) up to a maximum of 100 ml at a rate of 50 ml/min. The ice water was retained in the bladder for 1 min. A positive IWT was defined as presence of uninhibited detrusor contraction evoked by cold saline, associated with urgency or with fluid expulsion (Fig 1). In addition, the patients were asked to report pain and/or cold sensation in the bladder during the test. The "Pain Scores" were recorded on a visual analogue scale before urodynamics (baseline), during routine urodynamics (at 100 ml filling and capacity) and the IWT. The mean temperature of the ice water was measured at entry and exit and was found to be 1.4 and 19.8°C respectively.

For statistical analysis, the Fisher's exact P test was used for inter-group comparisons. The Wilcoxon signed-rank test was used to analyse the changes in the VAS scores at baseline, during the IWT and cystometrogram (CMG). A P value of less than 0.05 was considered to be statistically significant. The Stata version 8 program was used for statistical analyses.

## Results

### Bladder Cooling Reflex

Six of the 22 IDO patients had a positive IWT (Table 1). The IWT was positive in all four NDO patients. The mean (range) rise in detrusor pressure during the positive IWT was 44.7 (21–78) cm. H_2_O. None of the control or PBS patients demonstrated a positive IWT (Table 1).

### Cold sensation

All the patients reported cold sensation in the urethra during filling and subsequent voiding. However, only six (9.3%) of the patients reported cold sensation in the suprapubic region (1 NDO, 2 IDO, 3 PBS). Although, this incidence was higher in the PBS group (3/17, 17.6%) compared to the controls (0/21, 0%), the difference in the risk was 0.17 and missed statistical significance (P = 0.08). No significant association was observed of this cold sensation with a positive IWT or pain.

### Pain

**Figure 2:**
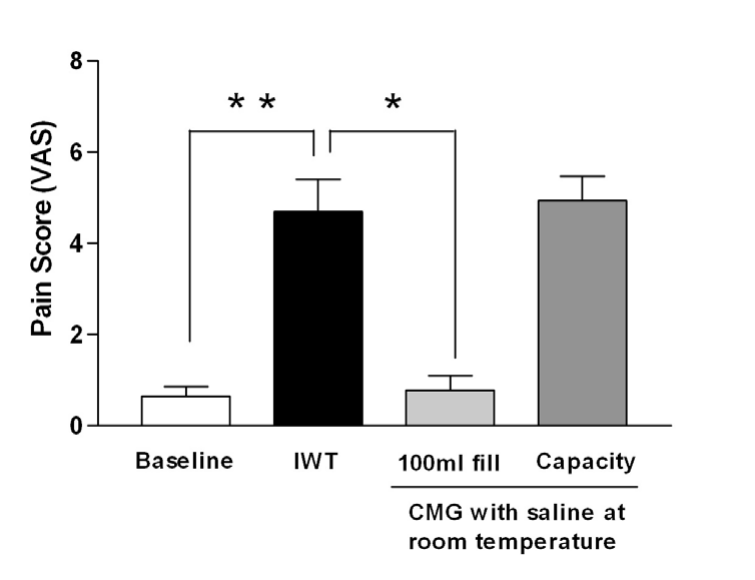
**Pain scores in PBS patients at baseline, IWT and CMG**. Bar chart showing the Pain Scores (VAS) at baseline, during the Ice Water test (IWT), filling to 100 ml with saline at room temperature, and filling to bladder capacity during routine urodynamics, in PBS patients (n = 17). Significantly higher pain scores were observed during ice water instillation compared to the baseline (P = 0.0002**) or equivalent amount of bladder filling (100 mls) with saline at room temperature (P = 0.015*), and were similar to pain scores on filling to bladder capacity.

Pain associated with bladder filling to capacity during urodynamics was reported by 16 of the 17 PBS patients (94.1%). This pain was described as sharp, generally in the suprapubic region. Six of the patients also reported pain in the perineum, and pain was referred to the back/sacral area in one patient. The Pain score at maximum cystometric capacity (MCC) was significantly higher compared to the baseline scores (Pain Scores, VAS (MCC), Mean ± SEM, 4.94 ± 0.53, VAS (Baseline), 0.64 ± 0.21, P = 0.0005) (Fig 2). The median difference from baseline was 5 and the 95% confidence interval for the difference was from 3.25 to 6.33. The pain was relieved by voiding and the VAS returned to baseline in all but one patient.

Thirteen of 17 PBS (76.5%) patients reported pain during the IWT. The pain was similar in character (sharp) and distribution to the pain seen at maximum cystometric capacity. Pain Scores were significantly higher during IWT compared to baseline scores (Pain Scores, VAS (IWT), Mean ± SEM, 4.71 ± 0.69, VAS (Baseline), 0.64 ± 0.21, P = 0.0002) (Fig. 2). The median difference from baseline was 4 and the 95% confidence interval for the difference was from 2.58 to 5.53. The Pain score during IWT i.e. 100 mls ice water instillation was also compared with equivalent 100 mls filling with saline at room temperature VAS (CMG 100 ml). The pain score with ice water instillation was significantly higher compared to pain scores using saline at room temperature (Pain Scores, VAS (IWT), Mean ± SEM, 4.71 ± 0.69, VAS (CMG 100 ml), 0.77 ± 0.32, P = 0.015) (Fig 2). The median difference was 5, the 95% confidence interval for the difference was from 1.9 to 5.7. The pain following IWT was relieved by voiding in 10/13 patients. The pain persisted following voiding in 3/13 patients, and required simple analgesia for symptom relief.

None of the control or overactive (NDO/IDO) patients reported pain during IWT or during routine urodynamics.

## Discussion

**Figure 3:**
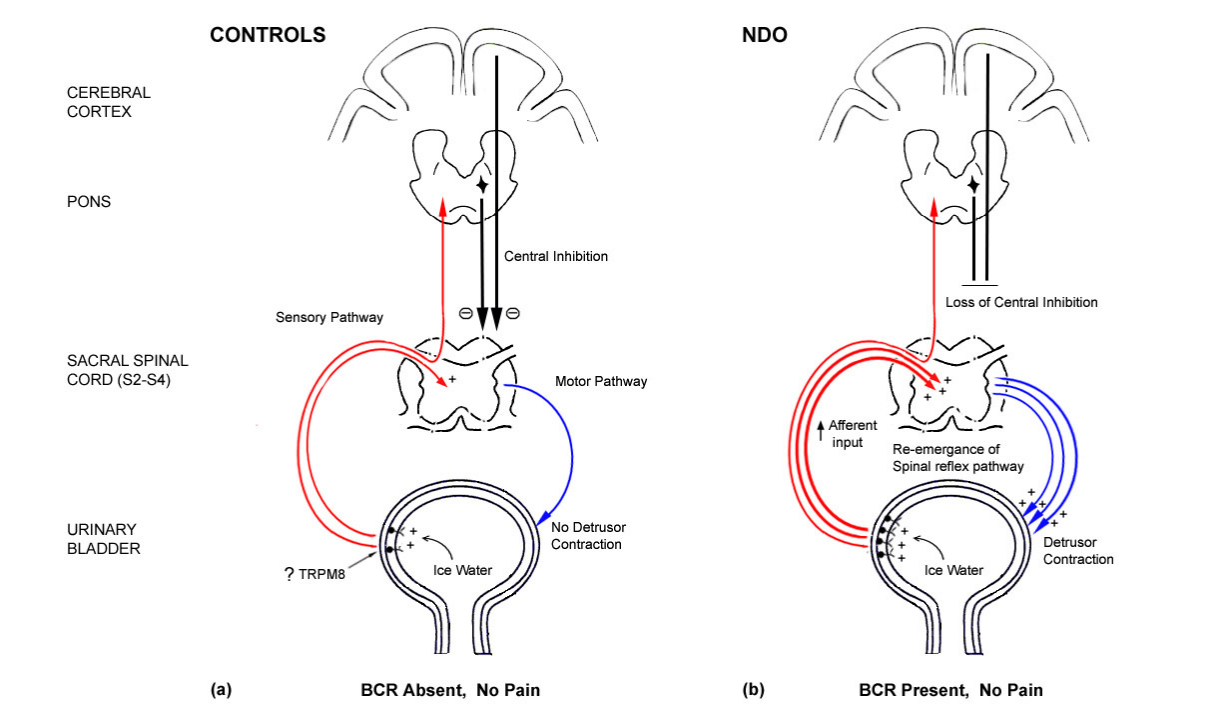
**Mechanisms of the BCR in control and neurogenic (NDO) bladders**. Diagram of proposed mechanisms of the bladder cooling reflex and pain in patient sub-groups during the ice water test (IWT). In neurologically normal adults and older children (> 4 years), the segmental spinal reflex pathway is normally inhibited by descending signals from higher centres (**a**). There is loss of central inhibition and increased afferent activation in neurogenic detrusor overactivity (NDO) patients (**b**), resulting in a positive IWT.

**Figure 4:**
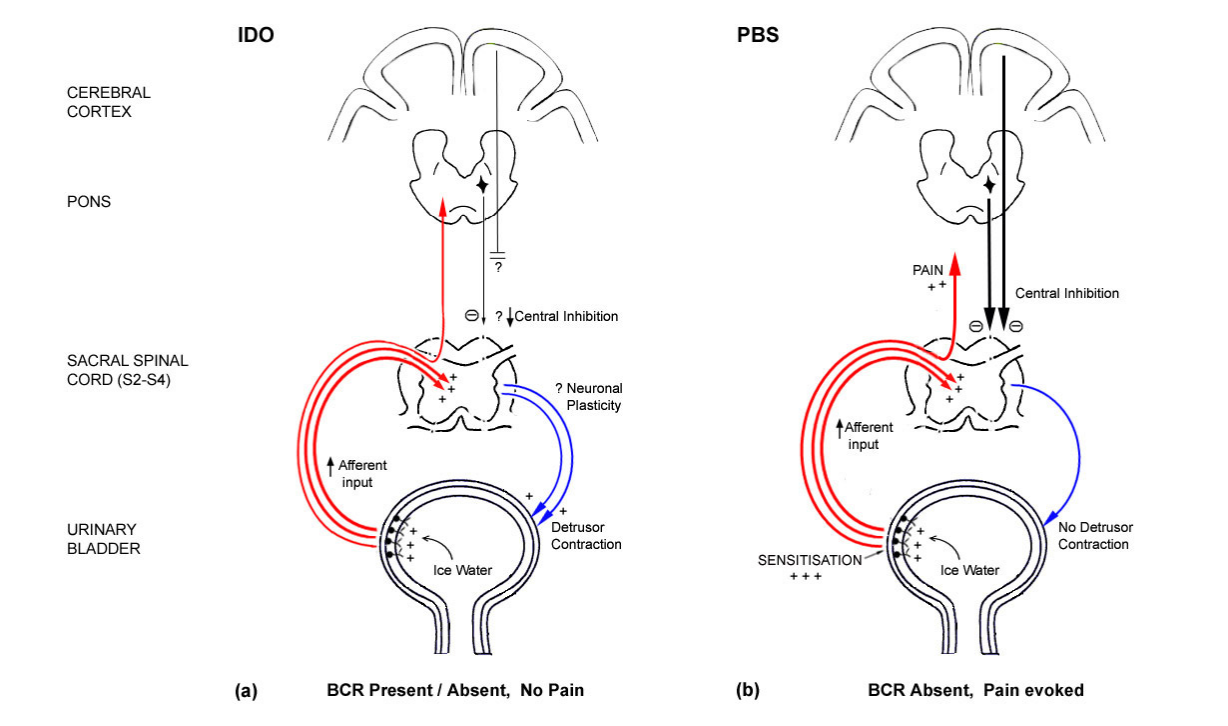
**Mechanisms of the BCR and pain in overactive (IDO) and painful (PBS) bladders**. Diagram of proposed mechanisms of the bladder cooling reflex and pain in patient sub-groups during the ice water test (IWT). In idiopathic detrusor overactivity (IDO) patients (**a**), a positive (IWT) may be a result of a loss of central inhibition and increased afferent activation, while the Pain evoked in Painful Bladder Syndrome (PBS) patients (**b**) may suggest sensitisation of increased afferents but preserved central inhibition.

This is the first study to evaluate the outcome of ice water test in patients with painful bladder syndrome, in comparison with overactive bladder disorders and controls. None of our controls or PBS patients had a positive IWT, indicating the inhibitory signals from higher centres being preserved, result in a negative IWT (Fig 3a, 4b). All our NDO patients demonstrated a positive IWT, which is in agreement with studies that demonstrate a positive test in up to 90% of patients with upper motor neuron lesions [13,14]. The BCR thus reflects the loss of central inhibition in DO, analogous to the positive Babinsky sign in CNS pyramidal lesions (Fig 3b). One-fourth (27.3%) of our IDO patients showed a positive IWT. In a previous study, Petersen and colleagues [15] noted a positive bladder cooling test in 15% of patients with unstable detrusor and no known neurological disease. A positive IWT has also been observed in 27–47% detrusor overactivity secondary to bladder outlet obstruction (BOO) [16,17]. Steers and De Groat [18] have suggested that obstruction produces neural plasticity, presumably from hypertrophy and increased production of nerve growth factor, resulting in a spinal reflex which may contribute to detrusor dysfunction. Similar increase in NGF levels have also been found in bladder of patients with IDO [19,20]. Furthermore, it has been observed that patients with IDO and positive IWT later developed overt signs of neurological diseases during long-term follow-up [4]. In this study, patients with evidence of BOO on urodynamics were excluded. Thus, the positive IWT seen in our IDO patients may be a result of neural plasticity, with associated decreased central inhibition as a result of occult neurological disease (Fig 4a).

Opinion varies with respect to thermal perception from the bladder mucosa. Some investigators [21] have concluded that the bladder mucosa can distinguish between hot and cold fluids, while others have report that thermal sensation is perceived exclusively by urethral receptors and not by receptors in the bladder mucosa [22,23]. In our study, all patients undergoing the IWT described a cold sensation in the urethra, indicating the presence of thermal receptors. Cold sensation in the suprapubic region was reported during the IWT by 6 patients, presumably referred from the bladder or urethra. We noted no significant association of this finding with a positive IWT or pain.

The most interesting finding of this study was the report of pain during intravesical instillation of ice water by PBS patients but not by other groups. The BCR is thought to be triggered by TRPM8 receptors in the bladder, with afferent signals relayed by unmyelinated fibres [1]. In a previous study [6], we demonstrated increased expression of TRPM8-immunoreactive nerve fibres in PBS bladder biopsies, with a significant correlation of these fibres with pain. An increase in TRPM8 transcripts has also been shown in the bladder of rats with spinal cord injury [24]. The report of pain in PBS, but not in DO patients, may indicate peripheral and/or central sensitisation in PBS; hence sensory symptoms are evoked (Fig 4b). The bladder cooling reflex thus indicates hyper-reflexia, not pain (hence this reflex is presents in normal babies, and reappears after CNS lesions in patients who do not report bladder pain). It would be of interest to perform the IWT test in patients with DO and inter-current bladder infection.

Significantly higher pain scores were seen in the PBS patients during the ice water test and at capacity during urodynamics, compared to baseline scores prior to urodynamics. One of the limitations of the study was that the order of urodynamic investigation (normal saline at room temperature vs cold saline) was not randomized. However, the hyperalgesia to bladder filling in PBS has been demonstrated in previous studies [25]. Altered visceral sensations from the urinary bladder (i.e., pain at bladder filling) that accompany PBS may be the consequence of many factors, including changes in the properties of peripheral bladder afferents, which respond in an exaggerated manner to normally innocuous stimuli (allodynia) [26]. The key novel finding of the study was the significantly higher pain scores in PBS patients during ice water instillation compared to the baseline, or equivalent amount of bladder filling (100 mls) with saline at room temperature. Cold sensitive nociceptors are normally activated by temperatures below 20°C [1]. Thus, the observation of pain during ice water instillation may suggest that the cold sensitive nociceptors may be sensitised in PBS, hence the pain. TRPM8 is activated at about 25°C and down to 10°C, and may be modulated or sensitised in bladder afferents to mediate these effects; alternative molecular mechanisms, that do not involve TRPM8, are also possible.

Clinical trials in PBS/IC have thus far used visual analogue scales for pain rating, and have lacked well defined tools for evaluation of pathophysiologic mechanisms that produce pain. The ice water-induced bladder pain may prove to be a useful marker for recruitment and assessment of efficacy in PBS clinical trials, particularly for antagonists of TRPM8. This may also apply to other targets expressed by polymodal small nerve fibres, even TRPV1. The test is simple, rapid and minimally-invasive.

## Conclusion

The pain elicited by ice water instillation in patients with Painful Bladder Syndrome suggests peripheral and/or central sensitisation, evoking pain symptoms without reflex detrusor contractions. The ice water test may be a useful marker for clinical trials in PBS, and to differentiate this condition from overactive bladder disorders.

## List of abbreviations

BCR : Bladder cooling reflex

BOO : Bladder outlet obstruction

IDO : Idiopathic detrusor overactivity

IWT : Ice water test

NDO : Neurogenic detrusor overactivity

PBS : Painful bladder syndrome

## Competing interests

The author(s) declare that they have no competing interests.

## Authors' contributions

GM was involved in clinical assessment of patients, performed the ice water test, analysis of data and writing the manuscript. JW performed the urodynamics and IWT, analysis of data and helped writing the manuscript. IPC, CB and SKA helped conceive the study, with interpretation of the data, and writing the manuscript. PA conceived the study and participated in its design and coordination, interpretation and completion of the manuscript. All authors read and approved the manuscript.

## Pre-publication history

The pre-publication history for this paper can be accessed here:



## Figures and Tables

**Table 1:** Summary of BCR results in controls and bladder disorders

**Diagnosis**	**No. of cases**	**IWT Positive**	**Cold sensation in bladder during ice water filling**	**Pain during ice water filling**
**Control**	**21**	**0 **(0%)	**0 **(0%)	**0 **(0%)
**NDO**	**4**	**4 **(100%)	**1 **(25%)	**0 **(0%)
**IDO**	**22**	**6 **(27.3%)	**2 **(9.1%)	**0 **(0%)
**PBS**	**17**	**0 **(0%)	**3 **(18%)	**13 **(76.5%)

Table showing the incidence of a positive IWT, cold sensation in bladder and pain, during IWT in control, overactive (NDO/IDO) and painful bladder patient sub-groups.
